# A head-to-head comparison of the validity and predictive ability for health outcomes of diagnosis versus medication-based comorbidity indices

**DOI:** 10.1007/s40520-025-03073-w

**Published:** 2025-05-27

**Authors:** Moritz Platen, Maresa Buchholz, Anika Rädke, Fabian Kleinke, Annelie Scharf, Michelle Pfaff, Audrey Iskandar, Neeltje van den Berg, Wolfgang Hoffmann, Bernhard Michalowsky

**Affiliations:** 1 German Center for Neurodegenerative Diseases (DZNE), site Rostock/Greifswald, Ellernholzstrasse 1-2, D-17489 Greifswald, Germany; 2 Institute for Community Medicine, Section Epidemiology of Health Care and Community Health, University Medicine Greifswald (UMG), Ellernholzstrasse 1-2, D-17489 Greifswald, Germany

**Keywords:** Multimorbidity, Comorbidity indices, Risk adjustment, Patient-reported outcomes, Predictive models

## Abstract

**Background:**

Various comorbidity indices have been validated for individual health outcomes. However, systematic concurrent comparisons of multiple outcome measures in a single study remain relatively underrepresented but needed for practical decision support.

**Aims:**

To compare the performance of the Charlson Comorbidity Index (CCI) and Rx-Risk Comorbidity Index (Rx-Risk).

**Methods:**

Baseline and six-month follow-up data from *n* = 221 patients recruited in *n* = 70 practices were used. CCI and Rx-Risk scores were calculated using documented diagnoses and prescribed medications. Outcomes assessed were health-related quality of life (HRQoL, EQ-5D-5 L), functional impairment (B-ADL), cognitive decline (MMSE), and healthcare utilization (physician visits, hospitalizations). Indices performance was evaluated regarding agreement (Cohens Kappa (*k*)), known-groups validity (ANOVA, t-test), convergent validity (correlation coefficient (*r*_*s*_)) and predictive ability (R², Akaike information criterion (AIC)).

**Results:**

Patients were, on average, 80 years old, mostly female (55%), with 12 diagnoses and seven medications. Agreement between both indices was poor for all conditions except diabetes (*k* = 0.645) and chronic airway diseases (*k* = 0.486). Rx-Risk differed more in known groups, especially for HRQoL and hospitalizations, and showed stronger correlations with the EQ-5D index (*r*_*s*_, -0.215 vs. -0.134) and risk of hospitalization (*r*_*s*_, 0.145 vs. 0.128) than CCI. Rx-Risk, again, performed better in predicting the change of EQ-5D index (R², 30 vs. 28%) and all EQ-5D dimensions, functional (R², 55 vs. 52%) and cognitive decline (R², 47 vs. 46%) and physician consultations (AIC, 649.2 vs. 651.0), except for hospitalization (AIC, 149.2 vs. 147.1).

**Conclusions:**

Rx-Risk demonstrated slightly superior validity and predictive ability for HRQoL and healthcare utilization, making it a promising option for studies focused on these outcomes. However, limitations regarding functional and cognitive impairment suggest alternative instruments are needed.

**Supplementary Information:**

The online version contains supplementary material available at 10.1007/s40520-025-03073-w.

## Introduction

Multimorbidity or comorbidity is highly prevalent in the older population [1, 2] and is often associated with poor health-related quality of life (HRQoL) [3], mortality [4], loss of physical function [5], and adverse events such as hospitalizations [6], which also has health economic implications. Several comorbidity indices were developed to quantify the specific burden of chronic conditions, control for confounding and adjust for risks comparing health outcomes or predicting health changes [7–9]. However, there are differences in index performance across health outcomes and studies comparing them systematically and concurrently are remain relatively underrepresented.

Generally, a distinction is made between self-reported (e.g. Self-Administered Comorbidity Questionnaire [10]), diagnosis (e.g. Elixhauser comorbidity index [11]) and medication-based (e.g. Chronic Disease Score [12]) comorbidity indices. Self-reported multimorbidity measures perform better regarding HRQoL and function-related outcomes but simultaneously could be affected by patients’ subjective perception and recall bias [13–15]. Diagnosis-based metrics are better predictors of mortality and are characterized by easy operationalization due to the availability of data from medical records or reimbursement data but can be affected by coding biases and blind spots regarding the severity of the respective conditions [16–18]. Medication-based measures have been developed to overcome these deficits, providing reliable data due to their more systematic linkage to the underlying diseases and their better performance in predicting health outcomes [18, 19].

Several systematic reviews support choosing the best comorbidity index that fits the designs and methodologic needs [9, 19, 20]. But even though prior research has explored various comorbidity indices there is substantial heterogeneity and significant discrepancies in their performance, underlining the need for standardization in comorbidity measurement to gain more comparable results [4, 19, 21]. Additionally, large-scale studies increasingly aim to capture the multidimensionality of health by examining multiple outcomes– such as clinical, patient-reported, and health-economic measures– simultaneously within a single study design. However, previous validation studies do not take into account the aspect of examining several health-related outcomes within the same patient population. In particular, outcomes like subjective health perspectives (HRQoL), functional impairment regarding activities of daily living, cognitive decline, and healthcare utilization (e.g., physician visits, hospitalizations) provide complementary information on older adults’ health. Although established comorbidity indices aim at the holistic quantification of the chronic disease burden, it is difficult to adequately capture the interplay between physical, cognitive, and psychosocial factors. This poses specific methodical challenges and often conflicts with ambitions such as data minimization. Therefore, research is needed to systematically compare comorbidity indices across concurrent outcomes to guide researchers in selecting the most suitable comorbidity index for their studies.

Thus, the present study aims to evaluate the agreement, known-groups validity, convergent validity and predictive ability of the most frequently used diagnosis- (i.e. Charlson comorbidity index (CCI) [22]) and medication-based (i.e. Rx-Risk comorbidity index (Rx-Risk) [23]) index across multiple complementary outcomes reflecting clinical (functional impairment, cognitive decline), patient-reported (HRQoL) and health-economic (healthcare utilization) domains to provide decision support for future study conceptualization.

## Materials and methods

### Data source and study population

The present analysis was based on data from the InDePendent trial [24]. Patients were recruited in *n* = 70 physician practices who are members of one of five participating physician networks in Germany. A total of *n* = 471 patients met the eligibility criteria (DemTect < 9 or formally diagnosed with dementia, ≥ 70 years old, living at home) and provided written informed consent. The InDePendent trial was approved by several ethics committees (University Medical Center Greifswald (BB 144/20); the State Medical Council Brandenburg (AS 81(bB)/2020); the State Medical Council of Hesse (2020-2081-zvBO)). The recruitment started in January 2021 and ended in December 2022. The detailed design has been described elsewhere [24].

A total of *n* = 417 patients started the baseline assessment, and *n* = 54 dropped out after baseline, resulting in a sample of *n* = 363 patients who started the 6-month follow-up. Patients who completed the follow-up assessment were considered for the present analyses, resulting in a sample of *n* = 221 patients.

### Data assessment

Nurses conducted a comprehensive, standardized, computer-assisted interview at the participants’ homes at baseline and six months after, assessing patients’ sociodemographics (age, sex, living situation) and clinical variables. Additionally, ICD-10 (International Statistical Classification of Diseases and Related Health Problems, 10th revision) diagnoses listed in the general practitioner (GP) files were documented [25], and medication data comprising all regularly taken drugs were validated with medication lists provided by the treating GP or, if available, by the administering nursing service.

### Comorbidity scores

Both Charlson and Rx-Risk scores, which represent fundamentally different approaches to measuring multimorbidity for each patient were calculated using documented diagnoses and recorded medications obtained from the medical records of the treating GP. The currency of diagnoses and regular use of medications at the time of data collection (only baseline) were confirmed by GPs or the drug-administrating nursing services. The CCI was applied, representing the most commonly used diagnose-based comorbidity score considering 17 diseases (Supplementary Tables 1a) [22]. An updated coding algorithm based on the ICD-10 System for the CCI by Quan et al. [26] was used to calculate a weighted sum score, considering the presence of both the respective conditions and their severity. Comorbidity was further categorized as follows according to previous research [27]: no (score < 1), low (score = 1), high (score = 2–3) and very high (score > 3). As a proxy for the medication-based comorbidity index, the Rx-Risk was used comprising 46 comorbidities linked to their indicative Anatomical Therapeutic Chemical (ATC) codes and weighted differently based on their predictive validity for mortality in an elderly population to calculate an overall comorbidity score (Supplementary Tables 1b) [23]. The higher the score, the higher the degree of comorbidity. The Rx-Risk score was then divided into quartiles corresponding to the following categories in line with recent research [28]: no, low, high, and very high degree of comorbidity.

### Outcome measures

#### Health-related quality of life

The EQ-5D-5 L was collected to measure HRQoL [29, 30]. The widely used, generic, preference-based, multidimensional EQ-5D‑5 L comprises five dimensions (mobility, self-care, pain/discomfort, usual activities, and anxiety/depression) and five levels (no, slight, moderate, severe, and extreme problems). Additionally, the level sum score (LSS) were calculated and a visual analog scale (EQ-VAS) anchored at 0 (worst health) and 100 (best health) assessed self-reported HRQoL [29–31]. The EQ-5D-5 L responses were converted to health utility values using the German value set, anchored at 0 (death) and 1 (full health) [32].

#### Functional and cognitive impairment

Functional impairment was measured by the Bayer Activities of Daily Living Scale (B-ADL) [33], ranging from 1 (best functional status) to 10 (worst functional status). Cognitive function was assessed using the Mini-Mental State Examination (MMSE) [34], ranging from 0 to 30, whereas a higher score indicates better cognitive function.

#### Health resource use

Health resource use was assessed using the Questionnaire for Health-related Resources in Older People (FIMA) [35]. Detailed information about frequencies or quantities of used medical and formal care services was recorded during the mentioned standardized, computer-assisted interviews at the participants’ homes at baseline and six months after. Caregivers’ proxy ratings were captured to improve data validity and precision. Hospitalization in the past 6 months was assessed dichotomously (at least one vs. none), and GP use in the previous quarter was assessed as a count data (number of consultations).

### Statistical analyses

All statistical analyses were conducted with STATA/IC software, version 16 [36].

#### Agreement

The agreement between both indices for overlapping conditions (congestive heart failure, diabetes, gastric condition, liver disease, chronic airway disease, dementia, renal disease) was assessed by Cohens Kappa (*k*), whereby *k* > 0.40 indicates acceptable agreement [37].

#### Known-groups validity

Known-groups validity was evaluated to distinguish between the degree of comorbidity and different stages of HRQoL (EQ-5D-5 L index, dimensions, VAS), functional (B-ADL) and cognitive (MMSE) impairment, and health resource use (GP contacts and hospitalization) using one-way analyses of variance (> 2 groups) and t-tests (2 groups) or Pearson’s chi-squared test (sensitivity analyses). Moreover, different comorbidity categories were compared regarding the mentioned outcomes for sensitivity analyses.

#### Convergent validity

Convergent validity between the CCI and Rx-Risk scores and outcome variables was assessed using the Pearson correlation coefficient (*r*_*p*_, parametric variables), Spearman’s correlation coefficient (*r*_*s*_, nonparametric variables) and point-biserial correlation coefficient (*r*_*pb*_, binary variables). A correlation coefficient > 0.3 was determined as a moderate correlation [38].

#### Predictive ability

Multiple regression models were performed with the respective comorbidity index as a predictor of interest and each outcome six months after baseline as a dependent variable to test the predictive ability. Specifically, for continuous outcomes (EQ-5D-5 L VAS, index, LSS, BADL, and MMSE), general linear regression models were conducted. For the ordinal outcome (EQ-5D-5 L dimensions), ordinal logistic regression models were used. The count outcome (GP contacts) was analyzed using Poisson regression models, and the binary outcome (hospitalizations) was assessed using logistic regression models. Models were adjusted for age, sex, living situation, and the baseline value of each outcome and standard errors were clustered at the GP practice level. The predictive performance of the indices was determined by the coefficient R² for parametric variables or the Akaike information criterion (AIC) for nonparametric variables [39]. A higher R² and lower AIC indicate better model performances [39]. The evidence for a better fitting model is determined by AIC differences between the compared models as follows: 0–2 (weak), 2–6 (positive), 6–10 (strong) and > 10 (very strong) [40]. Furthermore, the receiver operating characteristic (ROC) curves and c statistic were computed in addition to the AIC for the risk of hospitalization. C statistics > 0.8 indicate excellent discrimination [41].

## Results

### Sample characteristics

Table 1 summarizes the patient characteristics.

**Table 1:** Patient characteristics

	Total sample (*n* = 221)	
***Demographics***		
Age, mean (SD)	79.9	(6.5)
Sex (female), n (%)	122	(55.2)
Living alone, n (%)	93	(42.1)
***Clinical characteristics***		
Cognitive status (MMSE), mean (SD)	18.6	(6.9)
Depression (GDS), mean (SD)	3.3	(2.7)
Functional impairment (B-ADL), mean (SD)	5.6	(2.2)
Number of diagnoses, mean (SD)	11.8	(10.2)
Charlson score, mean (SD)	2.4	(2.6)
Number of medications, mean (SD)	6.8	(3.6)
Rx-Risk score, mean (SD)	3.5	(3.4)

Abbreviations: *B-ADL* Bayer-Activities of Daily Living Scale, range 0–10, lower score indicates better performance; *GDS* Geriatric Depression Scale, sum score 0–15, score ≥ 6 indicates depression; *ICD* International Statistical Classification of Diseases and Related Health Problems; *MMSE* Mini-Mental State Examination, range 0–30, higher score indicates better cognitive function; *SD* standard deviation

### Agreement

The analysis revealed poor agreements between overlapped conditions, indicated by *k* < 0.4, except for diabetes (*k* = 0.645, CI 95% 0.529–0.762) and chronic airway diseases (*k* = 0.486, CI 95% 0.300–0.672). The CCI covers more identified cases than the Rx-Risk, particularly for diabetes (81% vs. 78%), chronic airways (69% vs. 46%), dementia (92% vs. 37%), and renal diseases (100% vs. 3%), but was inferior to the Rx-Risk for congestive heart failure (17% vs. 98%), gastric conditions (6% vs. 97%) and liver diseases (29% vs. 71%) (see Table 2).

**Table 2:** Comparison of CCI and Rx-Risk in identifying medical conditions (Agreement)

	Patients identified by						
	at least one index *n* (%)	both indices *n* (%)	CCI only *n* (%)	RxRisk Index only *n* (%)	CCI *n* (%)	RxRisk Index *n* (%)	k (95% CI)
Congestive heart failure	118 (100)	18 (15.3)	2 (1.7)	98 (83.1)	20 (17.0)	116 (98.3)	0.131 (0.060–0.201)
Diabetes	**72 (100)**	**42 (62.2)**	**16 (22.2)**	**14 (19.4)**	**58 (80.6)**	**56 (77.8)**	**0.645 (0.529–0.762)**
Gastric condition	73 (100)	2 (2.7)	2 (2.7)	69 (94.5)	4 (5.5)	71 (97.3)	0.020 (-0.037–0.077)
Liver disease	7 (100)	N/A	2 (28.6)	5 (71.4)	2 (28.6)	5 (71.4)	-0.013 (-0.027–0.0004)
Chronic airways disease	**35 (100)**	**13 (37.1)**	**11 (45.8)**	**11 (45.8)**	**24 (68.6)**	**24 (68.6)**	**0.486 (0.300–0.672)**
Dementia	160 (100)	50 (31.3)	97 (66.0)	13 (8.1)	147 (91.9)	63 (37.4)	0.128 (0.035–0.222)
Renal disease	39 (100)	1 (2.6)	38 (97.4)	1 (2.6)	39 (100)	1 (2.6)	0.042 (-0.038–0.1213)

Abbreviations: *CCI* Charlson comorbidity index; *CI* confidence interval; *k* Cohens Kappa; *Rx-Risk* Rx-Risk comorbidity index

Bold values indicate k > 0.40; acceptable agreement

### Known-groups validity

The CCI could not statistically significantly differentiate between the known groups. In contrast, Rx-Risk could distinguish between the tertiles of the EQ-5D index score, the VAS, and the different stages of problems encountered in mobility and anxiety/depression or hospitalizations. Different age groups, further EQ-5D dimensions, cognitive and functional impairment, and GP consultations could be differentiated neither by the CCI nor the Rx-Risk index (see Table 3). Sensitivity analyses using different levels of comorbidity of the CCI and Rx-Risk index could significantly discriminate the EQ-5D index score or LSS, respectively (see Supplementary Table 2).

**Table 3:** Comparing known-groups validity of CCI and Rx-Risk

	Comorbidity Index, *n* = 221			*p* value
	CCI (score) mean (SD)	*p* value	Rx-Risk (score) mean (SD)	
**Age**				
≤ 81 years old, *n* = 118	2.45 (2.84)	0.927	3.74 (3.31)	0.324
> 81 years old, *n* = 103	2.42 (2.23)		3.28 (3.54)	
**Health-related Quality of Life**				
EQ-5D-5 L VAS				
1st tercile (≤ 51), *n* = 75	2.67 (2.36)	0.450	4.44 (3.80)	**0.017**
2nd tercile (52–70), *n* = 90	2.18 (2.56)		3.06 (3.15)	
3rd tercile (≥ 71), *n* = 56	2.54 (2.83)		3.05 (3.09)	
EQ-5D-5 L Index score				
1st tercile (≤ 0.771), *n* = 74	2.81 (2.45)	0.271	4.35 (3.58)	**0.038**
2nd tercile (0.772–0.887), *n* = 75	2.15 (2.57)		3.09 (3.33)	
3rd tercile (≥ 0.888), *n* = 72	2.35 (2.67)		3.13 (3.23)	
EQ-5D-5 L Dimensions				
*Mobility*				
No problem, *n* = 64	2.14 (2.61)	0.078	3.27 (2.88)	**≤ 0.001**
Slight to moderate problems, *n* = 131	2.37 (2.46)		3.11 (3.42)	
Severe to extreme problems, *n* = 26	3.46 (2.79)		6.27 (3.50)	
*Self-care*				
No problem, *n* = 90	2.58 (2.77)	0.113	3.40 (3.37)	0.154
Slight to moderate problems, *n* = 109	2.14 (2.41)		3.36 (3.33)	
Severe to extreme problems, *n* = 22	3.32 (2.30)		4.86 (3.89)	
*Usual activities*				
No problem, *n* = 56	2.55 (3.00)	0.497	3.52 (3.64)	0.333
Slight to moderate problems, *n* = 135	2.29 (2.42)		3.34 (3.38)	
Severe to extreme problems, *n* = 30	2.87 (2.32)		4.37 (3.16)	
*Pain/ discomfort*				
No problem, *n* = 83	2.11 (2.19)	0.065	2.93 (3.36)	0.086
Slight to moderate problems, *n* = 126	2.51 (2.71)		3.80 (3.39)	
Severe to extreme problems, *n* = 12	3.92 (3.03)		4.75 (3.79)	
*Anxiety/depression*				
No problem, *n* = 115	2.41 (2.92)	0.987	3.22 (3.52)	**0.032**
Slight to moderate problems, *n* = 94	2.46 (2.19)		3.60 (3.12)	
Severe to extreme problems, *n* = 12	2.50 (1.68)		5.92 (4.08)	
**Functional impairment (B-ADL)**				
No problems, *n* = 41	2.85 (3.16)	0.424	3.05 (3.29)	0.507
Moderate problems, *n* = 81	2.47 (2.57)		3.46 (2.94)	
Severe problems, *n* = 99	2.23 (2.28)		3.78 (3.83)	
**Cognitive status (MMSE)**				
No hint for dementia, *n* = 20	3.20 (2.91)	0.145	3.00 (2.49)	0.760
Mild dementia, *n* = 98	2.10 (2.14)		3.53 (3.52)	
Moderate/Severe dementia, *n* = 103	2.60 (2.83)		3.62 (3.50)	
**Health resource utilization**				
GP consultations				
1 visit, *n* = 96	2.30 (2.71)	0.299	3.67 (3.44)	0.067
2 visits, *n* = 58	2.19 (2.11)		2.67 (3.10)	
≥ 3 visits, *n* = 67	2.84 (2.68)		4.06 (3.56)	
Hospitalization				
Yes, *n* = 50	3.04 (3.27)	0.057	4.44 ( 3.69)	**0.031**
No, *n* = 171	2.26 (2.30)		3.26 (3.30)	

Abbreviations: *B-ADL* Bayer-Activities of Daily Living Scale; *CCI* Charlson comorbidity index; *GP* general practitioner; *MMSE* Mini-Mental State Examination; *r* correlation coefficient; *Rx-Risk* Rx-Risk comorbidity index; *VAS* visual analog scale

### Convergent validity

The Rx-Risk demonstrated more often statistically significant (*p* < 0.05) correlations with the outcome measures and larger correlation coefficients than the CCI, especially with the EQ-5D index (*rp* -0.215 vs. -0.134) and the LSS (*rp* 0.194 vs. 0.115), several EQ-5D dimensions, such as pain and discomfort (*rs* 0.170 vs. 0.165), anxiety and depression (*rs* 0.144 vs. 0.073), and the risk of hospitalization (*rpb* 0.145 vs. 0.128). Only for the EQ-5D dimension of mobility is the Rx-Risk score inferior to the CCI score (*rs* 0.142 vs. 0.172). However, in line with the definition of a moderate correlation (*r* > 0.3), none of the correlation coefficients reached this threshold. There was no correlation between indices and the EQ-5D VAS, EQ-5D dimensions of self-care, usual activities, cognitive and functional impairment, or GP consultations (see Table 4).

**Table 4:**
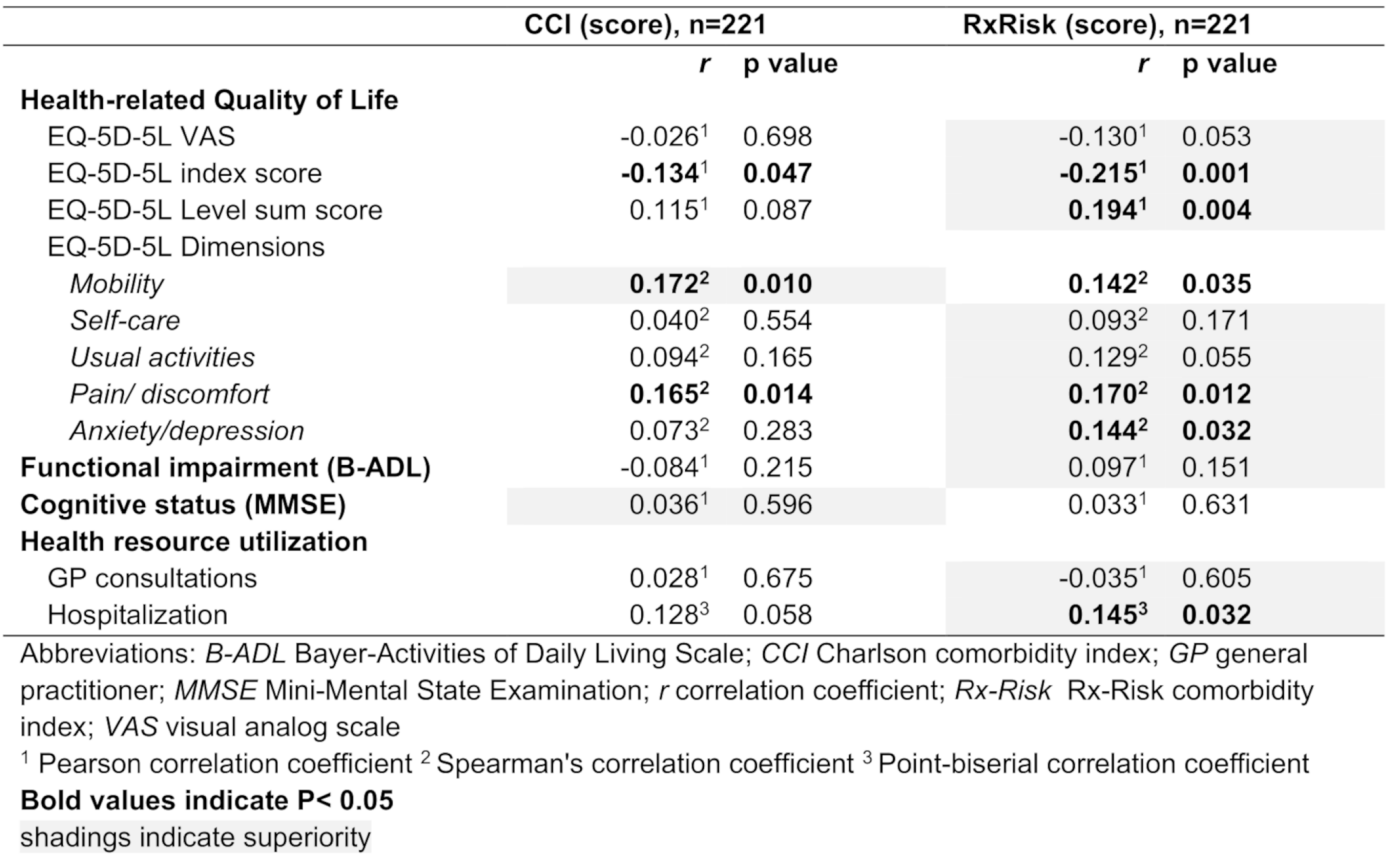
Comparing convergent validity of CCI and Rx-Risk

### Predictive ability

**Fig. 1:**
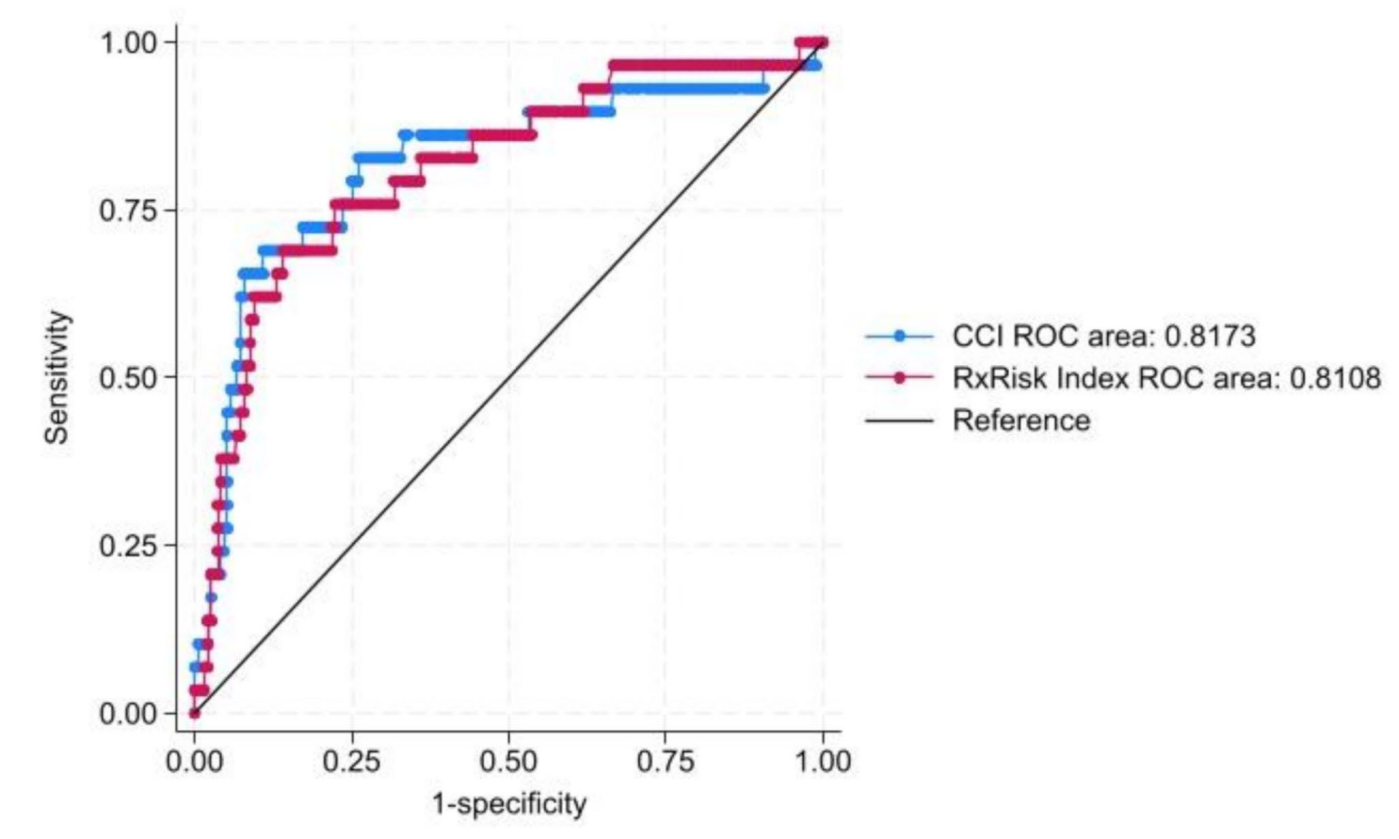
Comparing CCI and Rx-Risk using ROC Curves for predictability of hospitalization Abbreviations: *CCI* Charlson comorbidity index; *ROC* Receiver operating characteristics; *Rx-Risk* Rx-Risk comorbidity index

The Rx-Risk is superior to the CCI in predicting the proportion of variation (R²) for the association between baseline and the six-month follow-up on the EQ-5D index (R², 30 vs. 28%), LSS (R², 36 vs. 35%), and functional (R², 55 vs. 52%) and cognitive impairment (R², 47 vs. 46%). The previously defined threshold values for AIC differences ranged from weak (0–2) to positive (2–6). The Rx-Risk score performed better than the CCI in predicting all EQ-5D dimensions (mobility (AIC, 489.4 vs. 490.2); self-care (AIC, 552.5 vs. 557.5); usual activities (AIC, 580.6 vs. 584.4); pain/discomfort (AIC, 467.2 vs. 470.0); anxiety/depression (AIC, 446.9 vs. 447.3)) and GP consultations (AIC, 649.2 vs. 651.0). In contrast; the Rx-Risk was inferior to the CCI regarding the EQ-5D VAS (R², 9% vs. 10%) and risk of hospitalization (AIC, 149.2 vs. 147.1). Additionally, the c statistic for risk of hospitalization indicated that both indices exhibited excellent (i.e. c statistics > 0.8) discrimination (ROC area, CCI: 0.811 vs. Rx-Risk: 0.817) (see Table 5; Fig. 1).

**Table 5:**
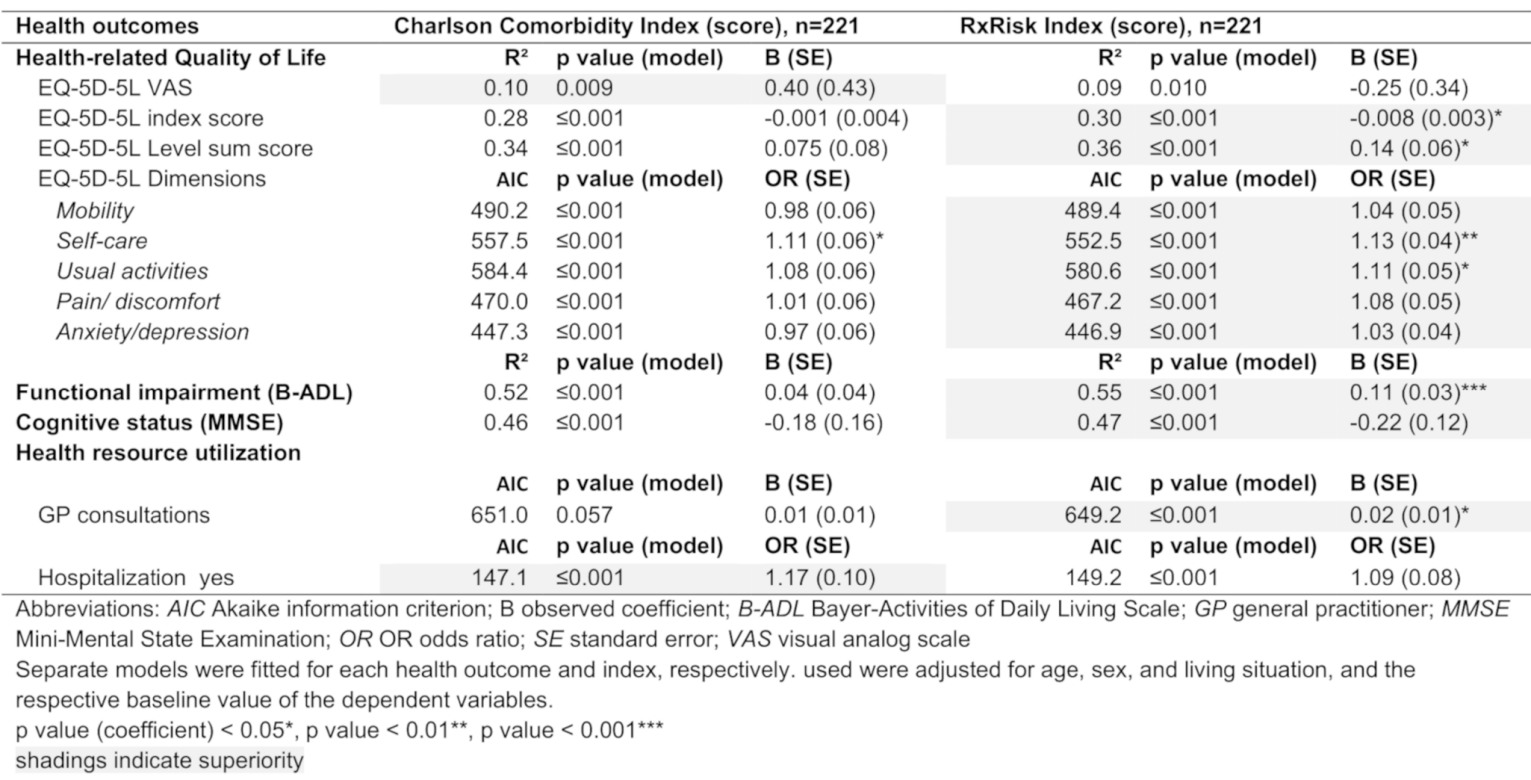
Comparing the predictive ability of CCI and Rx-Risk

## Discussion

This study confirmed that diagnosis-based (CCI) and medication-based (Rx-Risk) indices could tremendously differ in capturing chronic conditions, overlapping in only six chronic conditions with mainly poor agreement. Overall, the Rx-Risk was superior to the CCI in terms of known-groups validity and slightly superior regarding convergent validity and predictive ability. Differences favouring the CCI were insufficient to outperform the Rx-Risk noticeably.

Previously published studies have shown that the medication- and diagnosis-based indices differ significantly in detecting diseases covered by both, indicating a general poor agreement except for diabetes, which aligns with the present findings [42–44]. Biases and underestimation may accompany this lack of agreement. For dementia, the CCI detects 92%, and the Rx-Risk only 37% of cases identified as the sum of both instruments, indicating an underestimation of cases using the Rx-Risk in the German population. The underestimation problem becomes more evident, considering that even the CCI identified only 67% of the patients included in the present study, addressing the problem of underdiagnosing dementia in primary care, which should be considered when choosing the right instrument depending on specific populations to avoid potential biases [45, 46].

The Rx-Risk significantly outperforms the CCI in terms of the known-groups validity. Known groups of HRQoL (EQ-5D-5 L index score and VAS terciles), of experienced problems in the dimensions of mobility and anxiety/depression, and of hospitalizations differed considerably by the Rx-Risk index score. Importantly, when looking at the observed changes measured by the EQ-5D-5 L, mean changes particularly of the EQ-5D-5 L index terciles were higher than the minimal clinically important differences of 0.05 in chronic conditions [47]. However, known groups of age, other EQ-5D dimensions, cognitive and functional impairments, and GP visits could not be considerably delimited. Although the CCI is commonly applied to various outcomes in health services and clinical research, its inferior discriminative ability regarding these outcomes could be explained by the fact that it was initially conceptualized to predict mortality. In contrast, the Rx-Risk was updated and adjusted also for health economic outcomes, such as healthcare costs [19]. Applying both indices to the cognitive and functional impairment could be misleading. Although the indices cover multiple conditions that can potentially impact cognitive and functional decline, the instruments used could not reflect the different stages of dementia, which should be considered for trials conceptualization.

Regarding the convergent validity, several studies underline comparatively poor correlations between the respective instruments, especially HRQoL, and a slight inferiority of the CCI compared to medication-based scores [18, 48]. Consequently, these studies advise against its use for research examining HRQoL. This aligns with the present analysis, indicating only low correlations and a slight superiority of the Rx-Risk instrument over the CCI for HRQoL. Additionally, Beloosesky et al. [49] found at least correlations for the CCI with functional and cognitive impairment, whereas neither index exhibits significant results in the present analysis. However, participants were less cognitively and functionally impaired than in the present sample, which may suggest saturation effects, indicating insufficient variations at advanced levels of impairment and making additional effects unlikely to be observed [49]. Since none of the correlation coefficients reached the threshold of > 0.3 for moderate correlation, indicating overall poor correlations the practical implications of these correlations should be interpreted with caution, as the small differences in correlation coefficients may not translate into meaningful differences in patient care. The findings underline that the choice of the proper instrument depends not only on the target outcomes but also on the characteristics of the targeted patient populations. Therefore, using both indices for aged, functionally, and cognitively impaired patients is not recommended.

The multivariate analyses, predicting future values of the respective outcomes, confirmed the slight superiority of the medication-based Rx-Risk index compared to the diagnosis-based CCI. It is important to emphasize that despite the statistical superiority, the observed differences are relatively small; hence, the choice between indices should also consider contextual and practical aspects beyond numerical performance alone. Although both indices were initially not targeted on HRQoL, both were significant predictors for HRQoL change, with a slightly better predictive ability of the Rx-Risk index. The inferiority of the CCI was expected and underlined by several studies evaluating the performance of various indices in predicting HRQoL [48, 50, 51]. Additionally, Sasseville et al. [52] found a higher specificity of the medication-based compared to the diagnosis-based index, demonstrating poorer HRQoL according to the EQ-5D for increased multimorbidity. Despite the present findings indicating the suitability of the Rx-Risk for predicting HRQoL changes, further evidence from validation in appropriate populations is required for a final assessment and recommendation for use.

While the present study shows promising results for both instruments in predicting functional and cognitive impairment, with a slight superiority of the Rx-Risk instrument, recently published studies have shown that medication- and diagnosis-based indices, including the Rx-Risk and CCI, are either equivalent or show only poor discrimination, predicting especially functional impairment [52, 53]. Thus, the present results should also be used cautiously. Modified and validated instruments adapted to patients with cognitive impairment have been suggested to better predict cognitive status (e.g. the RxDx-Dementia Risk Index [54]).

In contrast to HRQoL, functional and cognitive impairment, in our study, the CCI performed better than the Rx-Risk regarding hospitalizations, which is inconsistent with previously published evidence. Wallace et al. [53] compared five diagnosis- and medication-based indices and found that the discrimination for all measures was poor, and all instruments were equivalent in predictive ability. However, the present results show excellent discrimination between patients at higher risk for hospitalizations and those at lower risk by Rx-Risk and CCI. Dominick et al. [55] exhibited the superiority of the Rx-Risk towards the CCI for physician visits and hospitalization in osteoarthritis patients. While the present results for physician visits were confirmed, those for hospitalizations were the opposite. Several studies suggest uncertainties in various instruments to predict hospitalizations, as emphasized by Radomski et al. [56], elaborating that the different comorbidity indices could drastically affect the direction of effect in outcomes. Additionally, Borson et al. [21] pointed out that different instruments adjusted for comorbidity are not equivalent and that dementia magnifies these discrepancies, which could also apply to the present sample. A standardized, valid, and reliable instrument adapted to dementia is needed to overcome these uncertainties.

### Limitations

Data were obtained from a small sample of German community-dwelling primary care patients, potentially limiting the generalizability of the presented results for in-patient or institutionalized patients. Participants included could therefore tend to have a lower degree of comorbidity. Moreover, the small sample size increases the risk of a Type II error (i.e., insufficient statistical power to detect true effects), which may limit the interpretation of the results. However, there are no standard criteria for calculating the sample size for this type of study. Concerning the psychometric validation, Anthoine et al. [57] revealed that the median sample size of 114 randomly selected validation studies was 207. As a rule of thumb, Rouquette et al. [58] demonstrated that a minimum of 300 subjects is generally acceptable for a validation study. The present study is based on a sample size of 221 patients. Therefore, a post hoc power analysis (Supplementary Table 3) was conducted for correlation and regression analyses indicating insufficient statistical power to detect true effects regarding the correlation analysis or convergent validity for all outcomes except for the EQ-5D-5 L index score and LSS. In contrast, the post hoc power analysis for the models that assessed the predictive ability achieved sufficient power for each outcome except for the number of GP consultations. Nevertheless, future studies with larger, more diverse populations are necessary to confirm and extend the present findings and to ensure sufficient power in discriminating relevant outcomes. Patients without formal dementia diagnosis at the time of study enrollment were screened for dementia, which could lead to false-positive screening results and consequently to a formal diagnosis by the treating GP that may not be valid in all cases. Both examined comorbidity indices, the Rx-Risk and CCI, were initially targeted to predict outcomes in administrative data, which could reflect coding and accounting behaviour; thus, results derived from their use in primary collected data may differ. Furthermore, both indices assume that patients’ diseases are recognized or treated and that they therefore have access to healthcare, which can lead to underestimation and the mentioned bias in coding. Additionally, neither index has been validated specifically for the German population.

## Conclusion

Comparing the diagnosis-based CCI and medication-based Rx-Risk indices revealed tremendous differences. The two indices captured different chronic conditions with only some overlapping conditions and could not be used as mutual substitutes for each other. However, the Rx-Risk was superior in differentiating between known groups and predictive ability. The Rx-Risk index mainly performed better in predicting HRQoL changes over time.

However, caution is warranted due to the relatively small sample size, which may indicate insufficient statistical power to detect true effects. Particularly discrepancies in predicting health resource utilization and the limitations in assessing functional and cognitive impairment underscore the need for further research based on larger sample sizes. Alternative instruments may offer better predictions for the patient-reported outcomes, such as functional and cognitive status, and should, therefore, be used as covariates in prediction models instead. Thus, standardized, reliable instruments, particularly adapted to the underlying condition of interest, are needed to overcome uncertainties in predicting outcomes and changes in outcomes. A decision for or against a specific multimorbidity index should always consider the nature and the number of outcomes and the specific population planned for future studies.

## Electronic supplementary material

Below is the link to the electronic supplementary material.

Supplementary Material 1

Supplementary Material 2

## Data Availability

No datasets were generated or analysed during the current study.
