# Supplementary material for: A head-to-head comparison of the validity and predictive ability for health outcomes of diagnosis versus medication-based comorbidity indices
Source: Aging Clin Exp Res. 2025 May 27;37(1):172. doi: 10.1007/s40520-025-03073-w (PMC12106559; doi:10.1007/s40520-025-03073-w)
Supplement: Supplementary file 2 — Supplementary Material 2 [file 40520_2025_3073_MOESM2_ESM.docx]

**Supplementary Table 1a** ICD-10 codes and weights used to calculate the Charlson Comorbidity Index

| **Diseases** | **ICD-10 coding according to Quan et al.** | **Weights according to Charlson et al.** |
| --- | --- | --- |
| Acute Myocardial Infarction | I21; I22; I25.2 | 1 |
| Congestive Heart Failure | I43; I50; I09.9; I11.0; I13.0; I13.2; I25.5; I42.0; I42.5; I42.6; I42.7; I42.8; I42.9; P29.0 | 1 |
| Peripheral Vascular Disease | I70; I71; I73.1; I73.8; I73.9; I77.1; I79.0; I79.2; K55.1; K55.8; K55.9; Z95.8; Z95.9 | 1 |
| Cerebrovascular Disease | G45; G46; I60; I61; I62; I63; I64; I65; I66; I67; I68; I69; H34.0 | 1 |
| Dementia | F00; F01; F02; F03; G30; F05.1; G31.1 | 1 |
| Chronic Pulmonary Disease | J40; J41; J42; J43; J44; J45; J46; J47; J60; J61; J62; J63; J64; J65; J66; J67; I27.8; I27.9; J68.4; J70.1; J70.3 | 1 |
| Rheumatologic Disease | M05; M32; M33; M34; M06; M31.5; M35.1; M35.3; M36.0 |  |
| Peptic Ulcer Disease | K25; K26; K27; K28 | 1 |
| Mild Liver Disease | B18; K73; K74; K70.0; K70.1; K70.2; K70.3; K70.9; K71.3; K71.4; K71.5; K71.7; K76.0; K76.2; K76.3; K76.4; K76.8; K76.9; Z94.4 | 1 |
| Diabetes without complications | E10.0; E10.1; E10.6; E10.8; E10.9; E11.0; E11.1; E11.6; E11.8; E11.9; E12.0; E12.1; E12.6; E12.8; E12.9; E13.0; E13.1; E13.6; E13.8; E13.9; E14.0; E14.1; E14.6; E14.8; E14.9 | 1 |
| Diabetes with chronic complications | E10.2; E10.3; E10.4; E10.5; E10.7; E11.2; E11.3; E11.4; E11.5; E11.7; E12.2; E12.3; E12.4; E12.5; E12.7; E13.2; E13.3; E13.4; E13.5; E13.7; E14.2; E14.3; E14.4; E14.5; E14.7 | 2 |
| Hemiplegia or Paraplegia | G81; G82; G04.1; G11.4; G80.1; G80.2; G83.0; G83.1; G83.2; G83.3; G83.4; G83.9 | 2 |
| Renal Disease | N18; N19; N05.2; N05.3; N05.4; N05.5; N05.6; N05.7; N25.0; I12.0; I13.1; N03.2; N03.3; N03.4; N03.5; N03.6; N03.7; Z49.0; Z49.1; Z49.2; Z94.0; Z99.2 | 2 |
| Cancer | C00; C01; C02; C03; C04; C05; C06; C07; C08; C09; C10; C11; C12; C13; C14; C15; C16; C17; C18; C19; C20; C21; C22; C23; C24; C25; C26; C30; C31; C32; C33; C34; C37; C38; C39; C40; C41; C43; C45; C46; C47; C48; C49; C50; C51; C52; C53; C54; C55; C56; C57; C58; C60; C61; C62; C63; C64; C65; C66; C67; C68; C69; C70; C71; C72; C73; C74; C75; C76; C81; C82; C83; C84; C85; C88; C90; C91; C92; C93; C94; C95; C96; C97 | 2 |
| Moderate or Severe Liver Disease | K70.4; K71.1; K72.1; K72.9; K76.5; K76.6; K76.7; I85.0; I85.9; I86.4; I98.2 | 3 |
| Metastatic Carcinoma | C77; C78; C79; C80 | 6 |
| AIDS/HIV | B20; B21; B22; B24 | 6 |
| Quan, H., et al., *Coding algorithms for defining comorbidities in ICD-9-CM and ICD-10 administrative data.* Med Care, 2005. **43**(11): p. 1130-9.  Charlson, M.E., et al., *A new method of classifying prognostic comorbidity in longitudinal studies: development and validation.* J Chronic Dis, 1987. **40**(5): p. 373-83. | | |

**Supplementary Table 1b** ATC codes and weights used to calculate the Rx-Risk comorbidity index

| **Diseases** | **ATC coding according to Pratt et al.** | **Weights according to Pratt et al.** |
| --- | --- | --- |
| Alcohol dependency | N07BB01; N07BB02; N07BB03; N07BB04; N07BB05; N07BB06; N07BB07 | 6 |
| Allergies | R01AC01; R01AC02; R01AC03; R01AC04; R01AC05; R01AC06; R01AC07; R01AC08; R01AC09; R01AC51; R01AD01; R01AD02; R01AD03; R01AD04; R01AD05; R01AD06; R06AD02; R06AD03; R06AD04; R06AD05; R06AD06; R06AD07; R06AD08; R06AD09; R06AD10; R06AD52; R06AD55; R06AE01; R06AE03; R06AE04; R06AE05; R06AE06; R06AE07; R06AE09; R06AE51; R06AE53; R06AE55; R06AX01; R06AX02; R06AX03; R06AX04; R06AX05; R06AX07; R06AX08; R06AX09; R06AX11; R06AX12; R06AX13; R06AX15; R06AX16; R06AX17; R06AX18; R06AX19; R06AX21; R06AX22; R06AX23; R06AX24; R06AX25; R06AX26; R06AX27; R06AB04 | -1 |
| Anticoagulants | B01AA03; B01AA04; B01AA07; B01AA08; B01AA09; B01AA10; B01AA11; B01AA12; B01AB01; B01AB02; B01AB04; B01AB05; B01AB06; B01AE07; B01AF01; B01AF02; B01AX05 | 1 |
| Antiplatelets | B01AC04; B01AC05; B01AC06; B01AC07; B01AC08; B01AC09; B01AC10; B01AC11; B01AC12; B01AC13; B01AC15; B01AC16; B01AC17; B01AC18; B01AC19; B01AC21; B01AC22; B01AC23; B01AC24; B01AC25; B01AC26; B01AC27; B01AC30 | 2 |
| Anxiety | N05BA01; N05BA02; N05BA03; N05BA04; N05BA05; N05BA06; N05BA07; N05BA08; N05BA09; N05BA10; N05BA11; N05BA12; N05BE01 | 1 |
| Arrhythmia | C01AA05; C01BA01; C01BA02; C01BA03; C01BA04; C01BA05; C01BA08; C01BA12; C01BA13; C01BA33; C01BA50; C01BA51; C01BA70; C01BA71; C01BB01; C01BB02; C01BB03; C01BB04; C01BC03; C01BC04; C01BC07; C01BC08; C01BC09; C01BD01; C07AA07 | 2 |
| Benign prostatic hyperplasia | G04CA01; G04CA02; G04CA03; G04CA04; G04CA05; G04CA51; G04CA52; G04CA53; G04CA54; G04CA55; G04CB01; G04CB02 | 0 |
| Bipolar disorder | N05AN01 | -1 |
| Chronic airways disease | R03AC02; R03AC03; R03AC04; R03AC05; R03AC06; R03AC07; R03AC08; R03AC09; R03AC10; R03AC11; R03AC12; R03AC13; R03AC14; R03AC15; R03AC16; R03AC17; R03AC18; R03AC19; R03AK01; R03AK02; R03AK03; R03AK04; R03AK05; R03AK06; R03AK07; R03AK08; R03AK09; R03AK10; R03AK11; R03AK12; R03AK13; R03AK14; R03AL01; R03AL02; R03AL03; R03AL04; R03AL05; R03AL06; R03AL07; R03AL08; R03AL09; R03AL10; R03AL11; R03AL12; R03BA01; R03BA02; R03BA03; R03BA04; R03BA05; R03BA06; R03BA07; R03BA08; R03BA09; R03BA19; R03BB01; R03BB02; R03BB03; R03BB04; R03BB05; R03BB06; R03BB07; R03BB08; R03BB54; R03BC01; R03BC03; R03BX01; R03CA02; R03CA51; R03CA52; R03CA53; R03CB01; R03CB02; R03CB03; R03CB51; R03CB53; R03CC02; R03CC03; R03CC04; R03CC05; R03CC06; R03CC07; R03CC08; R03CC09; R03CC10; R03CC11; R03CC12; R03CC13; R03CC14; R03CC15; R03CC53; R03CC54; R03CC63; R03DA01; R03DA02; R03DA03; R03DA04; R03DA05; R03DA06; R03DA07; R03DA08; R03DA09; R03DA10; R03DA11; R03DA12; R03DA20; R03DA50; R03DA51; R03DA52; R03DA53; R03DA54; R03DA55; R03DA57; R03DA63; R03DA73; R03DA74; R03DA82; R03DA90; R03DB01; R03DB02; R03DB03; R03DB04; R03DB05; R03DB06; R03DB12; R03DB13; R03DB20; R03DC01; R03DC02; R03DC03; R03DX05 | 2 |
| Congestive heart failure | C03DA02; C03DA03; C03DA04; C03DA05; C07AB07; C07AG02; C07AB12; C03DA04; C03CA01; C03CA02; C03CA03; C03CA04; C03CA05; C03CB01; C03CB02; C03CC01 & C09AA01; C09AA02; C09AA03; C09AA04; C09AA05; C09AA06; C09AA07; C09AA08; C09AA09; C09AA10; C09AA11; C09AA12; C09AA13; C09AA14; C09AA15; C09AA16 ; C09CA01; C09CA02; C09CA03; C09CA04; C09CA05; C09CA06; C09CA07; C09CA08; C09CA09; C09CA10; C09CA01; C09CA02; C09CA03; C09CA04; C09CA05; C09CA06; C09CA07; C09CA08; C09CA09; C09CA10 | 2 |
| Dementia | N06DA02; N06DA03; N06DA04; N06DX01 | 2 |
| Depression | N06AA01; N06AA02; N06AA03; N06AA04; N06AA05; N06AA06; N06AA07; N06AA08; N06AA09; N06AA10; N06AA11; N06AA12; N06AA13; N06AA14; N06AA15; N06AA16; N06AA17; N06AA18; N06AA19; N06AA20; N06AA21; N06AA23; N06AA25; N06AB02; N06AB03; N06AB04; N06AB05; N06AB06; N06AB07; N06AB08; N06AB09; N06AB10; N06AF01; N06AF02; N06AF03; N06AF04; N06AF05; N06AF06; N06AG02; N06AX03; N06AX04; N06AX05; N06AX06; N06AX07; N06AX08; N06AX09; N06AX10; N06AX11; N06AX13; N06AX14; N06AX15; N06AX16; N06AX17; N06AX18; N06AX21; N06AX22; N06AX23; N06AX24; N06AX26 | 2 |
| Diabetes | A10AB01; A10AB02; A10AB03; A10AB04; A10AB05; A10AB06; A10AB30; A10AC01; A10AC02; A10AC03; A10AC04; A10AC30; A10AD01; A10AD02; A10AD03; A10AD04; A10AD05; A10AD06; A10AD30; A10AE01; A10AE02; A10AE03; A10AE04; A10AE05; A10AE06; A10AE07; A10AE30; A10AE54; A10AE56; A10AF01; A10BA01; A10BA02; A10BA03; A10BB01; A10BB02; A10BB03; A10BB04; A10BB05; A10BB06; A10BB07; A10BB08; A10BB09; A10BB10; A10BB11; A10BB12; A10BB31; A10BC01; A10BD01; A10BD02; A10BD03; A10BD04; A10BD05; A10BD06; A10BD07; A10BD08; A10BD09; A10BD10; A10BD11; A10BD12; A10BD13; A10BD14; A10BD15; A10BD16; A10BD17; A10BD18; A10BD19; A10BD20; A10BD21; A10BD22; A10BD23; A10BD24; A10BD25; A10BD26; A10BD27; A10BD28; A10BD29; A10BD31; A10BF01; A10BF02; A10BF03; A10BG01; A10BG02; A10BG03; A10BG04; A10BH01; A10BH02; A10BH03; A10BH04; A10BH05; A10BH06; A10BH07; A10BH08; A10BH51; A10BH52; A10BJ01; A10BJ02; A10BJ03; A10BJ04; A10BJ05; A10BJ06; A10BJ07; A10BK01; A10BK02; A10BK03; A10BK04; A10BK05; A10BK06; A10BK07; A10BX02; A10BX03; A10BX05; A10BX06; A10BX08; A10BX15; A10BX16; A10BX17; A10BX18 | 2 |
| Epilepsy | N03AA01; N03AA02; N03AA03; N03AA04; N03AA05; N03AA30; N03AB01; N03AB02; N03AB03; N03AB04; N03AB05; N03AB52; N03AB54; N03AC01; N03AC02; N03AC03; N03AD01; N03AD02; N03AD03; N03AD51; N03AE01; N03AE02; N03AF01; N03AF02; N03AF03; N03AF04; N03AG01; N03AG02; N03AG03; N03AG04; N03AG05; N03AG06; N03AX03; N03AX07; N03AX09; N03AX10; N03AX11; N03AX13; N03AX14; N03AX15; N03AX17; N03AX18; N03AX19; N03AX21; N03AX22; N03AX23; N03AX24; N03AX25; N03AX26; N03AX27; N03AX30; N03AX31 | 0 |
| Glaucome | S01EA01; S01EA02; S01EA03; S01EA04; S01EA05; S01EA51; S01EA52; S01EB01; S01EB02; S01EB03; S01EC03; S01EC04; S01EC05; S01EC24; S01EC54; S01ED01; S01ED02; S01ED03; S01ED04; S01ED05; S01ED06; S01ED07; S01ED08; S01ED24; S01ED51; S01ED52; S01ED54; S01ED55; S01ED61; S01ED62; S01ED63; S01ED66; S01ED67; S01ED68; S01ED69; S01ED70; S01EE01; S01EE02; S01EE03; S01EE04; S01EE05; S01EE06; S01EE51; S01EX01; S01EX02; S01EX05; S01EX06; S01EX07 | 0 |
| Gastrooesophageal reflux disease | A02BA01; A02BA02; A02BA03; A02BA04; A02BA05; A02BA06; A02BA07; A02BA08; A02BA51; A02BA53; A02BB01; A02BB02; A02BC01; A02BC02; A02BC03; A02BC04; A02BC05; A02BC06; A02BC07; A02BC08; A02BC09; A02BC51; A02BC53; A02BC54; A02BD01; A02BD02; A02BD03; A02BD04; A02BD05; A02BD06; A02BD07; A02BD08; A02BD09; A02BD10; A02BD11; A02BD12; A02BD13; A02BD14; A02BD15; A02BD16; A02BD17; A02BX01; A02BX02; A02BX03; A02BX04; A02BX05 | 0 |
| Gout | M04AA01; M04AA02; M04AA03; M04AA51; M04AB01; M04AB02; M04AB03; M04AB04; M04AB05; M04AC01 | 1 |
| Hepatitis B | J05AF08; J05AF10; J05AF11 | NA |
| Hepatitis C | J05AB54; L03AB10; L03AB11; L03AB60; L03AB61; J05AE14; J05AE11; J05AE12; J05AX14; J05AX15; J05AX65; J05AB04 | NA |
| HIV | J05AE01; J05AE02; J05AE03; J05AE04; J05AE05; J05AE07; J05AE08; J05AE09; J05AE10; J05AF12; J05AF13; J05AG01; J05AG02; J05AG03; J05AG04; J05AG05; J05AR01; J05AR02; J05AR03; J05AR04; J05AR05; J05AR06; J05AR07; J05AR08; J05AR09; J05AR10; J05AR11; J05AR12; J05AR13; J05AR14; J05AR15; J05AR16; J05AR17; J05AR18; J05AR19; J05AR20; J05AR21; J05AR22; J05AR23; J05AR24; J05AR25; J05AR26; J05AR27; J05AX07; J05AX09; J05AX12; J05AF01; J05AF02; J05AF03; J05AF04; J05AF05; J05AF06; J05AF07; J05AF09 | 0 |
| Hyperkalaemia | V03AE01 | 4 |
| Hyperlipidaemia | A10BH03; C10AA01; C10AA02; C10AA03; C10AA04; C10AA05; C10AA06; C10AA07; C10AA08; C10AD01; C10AD02; C10AD03; C10AD04; C10AD05; C10AD06; C10AD08; C10AD09; C10AD52; C10AP03; C10AX01; C10AX02; C10AX03; C10AX05; C10AX06; C10AX07; C10AX08; C10AX09; C10AX10; C10AX11; C10AX12; C10AX13; C10AX14; C10AX15; C10AX16; C10AX17; C10AX18; C10AX19; C10AX21; C10AX22; C10BA01; C10BA02; C10BA03; C10BA04; C10BA05; C10BA06; C10BA07; C10BA08; C10BA09; C10BA10; C10BA11; C10BA12; C10BB01; C10BB02; C10BE11; C10BP03; C10BX01; C10BX02; C10BX03; C10BX04; C10BX05; C10BX06; C10BX07; C10BX08; C10BX09 | -1 |
| Hypertension | C03AA01; C03AA02; C03AA03; C03AA04; C03AA05; C03AA06; C03AA07; C03AA08; C03AA09; C03AA13; C03AB01; C03AB02; C03AB03; C03AB04; C03AB05; C03AB06; C03AB07; C03AB08; C03AB09; C03AH01; C03AH02; C03AX01; C03AX02; C03BA02; C03BA03; C03BA04; C03BA05; C03BA07; C03BA08; C03BA09; C03BA10; C03BA11; C03DB01; C03DB99; C03EA01; C09BA02; C09BA03; C09BA04; C09BA05; C09BA06; C09BA07; C09BA08; C09BA09; C09DA02; C09DA03; C09DA04; C09DA06; C09DA07; C09DA08; C02AB01; C02AB02; C02AC01; C02AC02; C02AC04; C02AC05; C02DB02; C02DB03; C02DB04; C03CA01; C03CA02; C03CA03; C03CA04; C03CA05; C03CB01; C03CB02; C03CC01; C09AA01; C09AA02; C09AA03; C09AA04; C09AA05; C09AA06; C09AA07; C09AA08; C09AA09; C09AA10; C09AA11; C09AA12; C09AA13; C09AA14; C09AA15; C09AA16 ; C09CA01; C09CA02; C09CA03; C09CA04; C09CA05; C09CA06; C09CA07; C09CA08; C09CA09; C09CA10; C09CA01; C09CA02; C09CA03; C09CA04; C09CA05; C09CA06; C09CA07; C09CA08; C09CA09; C09CA10 | -1 |
| Hyperthyroidism | H03BA02; H03BB01 | 2 |
| Hypothyroidism | H03AA01; H03AA02 | 0 |
| Irritable bowel syndrome | A07EC01; A07EC02; A07EC03; A07EC04; A07EA01; A07EA02; A07EA06; L04AA33 | 0 |
| Ischaemic heart disease: angina | C01DA02; C01DA04; C01DA05; C01DA07; C01DA08; C01DA09; C01DA13; C01DA14; C01DX16; C08EX02 | 2 |
| Ischaemic heart disease: hypertension | C07AA01; C07AA02; C07AA03; C07AA05; C07AA06; C07AA08; C07AA12; C07AA14; C07AA15; C07AA16; C07AA17; C07AA18; C07AA19; C07AA23; C07AA27; C07AA30; C07AA31; C07AB01; C07AB02; C07AG01; C08CA01; C08CA02; C08CA03; C08CA04; C08CA05; C08CA06; C08CA07; C08CA08; C08CA09; C08CA10; C08CA11; C08CA12; C08CA13; C08CA14; C08CA15; C08CA16; C08CA17; C08CA51; C08CA55; C08CX01; C08DA01; C08DA02; C08DA51; C08DA81; C08DB01; C09DB01; C09DB02; C09DB04; C09DX01; C09BB02; C09BB03; C09BB04; C09BB05; C09BB06; C09BB07; C09BB10; C07AB03; C09DX03; C10BX03 | -1 |
| Incontinence | G04BD01; G04BD02; G04BD03; G04BD04; G04BD05; G04BD06; G04BD07; G04BD08; G04BD09; G04BD10; G04BD11; G04BD12; G04BD13; G04BD14; G04BD15; G04BD19; G04BD20; G04BD21; G04BD59; G04BD66; G04BD69; G04BD71 | 0 |
| Inflammation/pain | M01AB01; M01AB02; M01AB03; M01AB04; M01AB05; M01AB06; M01AB07; M01AB08; M01AB09; M01AB10; M01AB11; M01AB12; M01AB13; M01AB14; M01AB15; M01AB16; M01AB17; M01AB19; M01AB51; M01AB55; M01AB68; M01AB69; M01AC01; M01AC02; M01AC03; M01AC04; M01AC05; M01AC06; M01AC56; M01AE01; M01AE02; M01AE03; M01AE04; M01AE05; M01AE06; M01AE07; M01AE08; M01AE09; M01AE10; M01AE11; M01AE12; M01AE13; M01AE14; M01AE15; M01AE16; M01AE17; M01AE18; M01AE20; M01AE51; M01AE52; M01AE53; M01AE56; M01AE57; M01AG01; M01AG02; M01AG03; M01AG04; M01AG06; M01AH01; M01AH02; M01AH03; M01AH04; M01AH05; M01AH06 | -1 |
| Liver failure | A06AD11; A07AA11 | 3 |
| Malignancies | L01AA01; L01AA02; L01AA03; L01AA05; L01AA06; L01AA07; L01AA08; L01AA09; L01AA10; L01AB01; L01AB02; L01AB03; L01AC01; L01AC02; L01AC03; L01AD01; L01AD02; L01AD03; L01AD04; L01AD05; L01AD06; L01AD07; L01AD08; L01AG01; L01AX01; L01AX02; L01AX03; L01AX04; L01BA01; L01BA03; L01BA04; L01BA05; L01BB02; L01BB03; L01BB04; L01BB05; L01BB06; L01BB07; L01BC01; L01BC02; L01BC03; L01BC04; L01BC05; L01BC06; L01BC07; L01BC08; L01BC09; L01BC52; L01BC53; L01BC58; L01BC59; L01BC63; L01BC73; L01CA01; L01CA02; L01CA03; L01CA04; L01CA05; L01CA06; L01CB01; L01CB02; L01CC01; L01CD01; L01CD02; L01CD03; L01CD04; L01CD51; L01CE01; L01CE02; L01CE03; L01CE04; L01CH01; L01CP01; L01CP02; L01CP50; L01CX01; L01DA01; L01DB01; L01DB02; L01DB03; L01DB04; L01DB05; L01DB06; L01DB07; L01DB08; L01DB09; L01DB10; L01DB11; L01DC01; L01DC02; L01DC03; L01DC04; L01EA01; L01EA02; L01EA03; L01EA04; L01EA05; L01EA06; L01EB01; L01EB02; L01EB03; L01EB04; L01EB05; L01EB06; L01EB07; L01EB08; L01EB09; L01EB10; L01EB11; L01EC01; L01EC02; L01EC03; L01ED01; L01ED02; L01ED03; L01ED04; L01ED05; L01EE01; L01EE02; L01EE03; L01EE04; L01EF01; L01EF02; L01EF03; L01EG01; L01EG02; L01EG03; L01EG04; L01EH01; L01EH02; L01EH03; L01EJ01; L01EJ02; L01EJ03; L01EJ04; L01EK01; L01EK02; L01EK03; L01EK04; L01EL01; L01EL02; L01EL03; L01EL04; L01EL05; L01EM01; L01EM02; L01EM03; L01EM04; L01EM05; L01EN01; L01EN02; L01EN03; L01EN04; L01EX01; L01EX02; L01EX03; L01EX04; L01EX05; L01EX06; L01EX07; L01EX08; L01EX09; L01EX10; L01EX11; L01EX12; L01EX13; L01EX14; L01EX15; L01EX17; L01EX18; L01EX19; L01EX21; L01EX22; L01EX23; L01EX24; L01EX25; L01FA01; L01FA02; L01FA03; L01FB01; L01FB02; L01FC01; L01FC02; L01FD01; L01FD02; L01FD03; L01FD04; L01FD05; L01FD06; L01FE01; L01FE02; L01FE03; L01FF01; L01FF02; L01FF03; L01FF04; L01FF05; L01FF06; L01FF07; L01FF08; L01FF09; L01FF10; L01FF11; L01FF12; L01FG01; L01FG02; L01FX01; L01FX02; L01FX03; L01FX04; L01FX05; L01FX06; L01FX07; L01FX08; L01FX09; L01FX10; L01FX11; L01FX12; L01FX13; L01FX14; L01FX15; L01FX16; L01FX17; L01FX18; L01FX19; L01FX20; L01FX21; L01FX22; L01FX23; L01FX24; L01FX25; L01FX28; L01FX29; L01FY01; L01FY02; L01FY03; L01XA01; L01XA02; L01XA03; L01XA04; L01XA05; L01XB01; L01XD01; L01XD03; L01XD04; L01XD05; L01XD06; L01XD07; L01XD10; L01XF01; L01XF02; L01XF03; L01XG01; L01XG02; L01XG03; L01XH01; L01XH02; L01XH03; L01XH04; L01XH05; L01XJ01; L01XJ02; L01XJ03; L01XK01; L01XK02; L01XK03; L01XK04; L01XK05; L01XK06; L01XK52; L01XL01; L01XL02; L01XL03; L01XL04; L01XL05; L01XL06; L01XL07; L01XL08; L01XL09; L01XL90; L01XX01; L01XX02; L01XX03; L01XX05; L01XX07; L01XX08; L01XX09; L01XX10; L01XX11; L01XX16; L01XX18; L01XX23; L01XX24; L01XX27; L01XX29; L01XX33; L01XX35; L01XX36; L01XX40; L01XX41 | 2 |
| Malnutrition | B05BA01; B05BA02; B05BA03; B05BA04; B05BA10 | 0 |
| Migraine | N02CA01; N02CA02; N02CA04; N02CA07; N02CA51; N02CA52; N02CA71; N02CA72; N02CB01; N02CC01; N02CC02; N02CC03; N02CC04; N02CC05; N02CC06; N02CC07; N02CC08; N02CC51; N02CD01; N02CD02; N02CD03; N02CD04; N02CD05; N02CD06; N02CD07; N02CH01; N02CH10; N02CH20; N02CP01; N02CP02; N02CP52; N02CX01 | -1 |
| Osteoporosis/Paget's | M05BA01; M05BA02; M05BA03; M05BA04; M05BA05; M05BA06; M05BA07; M05BA08; M05BB01; M05BB02; M05BB03; M05BB04; M05BB05; M05BX03; M05BX04; G03XC01; H05AA02 | -1 |
| Pain | N02AA01; N02AA02; N02AA03; N02AA04; N02AA05; N02AA08; N02AA10; N02AA11; N02AA51; N02AA53; N02AA55; N02AA56; N02AA57; N02AA58; N02AA59; N02AA79; N02AB01; N02AB02; N02AB03; N02AB07; N02AB52; N02AB72; N02AC01; N02AC03; N02AC04; N02AC05; N02AC06; N02AC52; N02AC54; N02AC74; N02AD01; N02AD02; N02AD51; N02AE01; N02AF01; N02AF02; N02AG01; N02AG02; N02AG03; N02AG04; N02AJ01; N02AJ02; N02AJ03; N02AJ05; N02AJ06; N02AJ07; N02AJ08; N02AJ09; N02AJ13; N02AJ14; N02AJ15; N02AJ16; N02AJ17; N02AJ18; N02AJ19; N02AJ22; N02AJ23; N02AX01; N02AX02; N02AX06; N02AX52; N02BE51 | 3 |
| Pancreatic insufficiency | A09AA02 | 0 |
| Parkinson's disease | N04AA01; N04AA02; N04AA03; N04AA04; N04AA05; N04AA08; N04AA09; N04AA10; N04AA11; N04AA12; N04AA13; N04AA14; N04AB01; N04AB02; N04AC01; N04AC30; N04AH20; N04BA01; N04BA03; N04BA04; N04BA05; N04BA06; N04BA07; N04BA10; N04BA11; N04BA13; N04BA14; N04BB01; N04BC01; N04BC02; N04BC03; N04BC04; N04BC05; N04BC06; N04BC07; N04BC08; N04BC09; N04BC10; N04BD01; N04BD02; N04BD03; N04BX01; N04BX02 | 3 |
| Psoriasis | D05AA01; D05AA02; D05AA50; D05AA51; D05AA52; D05BB01; D05BB02; D05AX02; D05AC01; D05AC51; D05AX52 |  |
| Psychotic illness | N05AA01; N05AA02; N05AA03; N05AA04; N05AA05; N05AA06; N05AA07; N05AB01; N05AB02; N05AB06; N05AB07; N05AB08; N05AB09; N05AB10; N05AB13; N05AC01; N05AC02; N05AC03; N05AC04; N05AD01; N05AD02; N05AD03; N05AD04; N05AD05; N05AD06; N05AD07; N05AD08; N05AD09; N05AD10; N05AE01; N05AE02; N05AE03; N05AE04; N05AE05; N05AF01; N05AF02; N05AF03; N05AF04; N05AF05; N05AG01; N05AG02; N05AG03; N05AH01; N05AH02; N05AH03; N05AH04; N05AH05; N05AH06; N05AH53; N05AL01; N05AL02; N05AL03; N05AL04; N05AL05; N05AL06; N05AL07; N05AX07; N05AX08; N05AX10; N05AX11; N05AX12; N05AX13 | 6 |
| Pulmonary hypertension | C02KX01; C02KX02; C02KX03; C02KX04; C02KX05 | 6 |
| Renal disease | B03XA01; B03XA02; B03XA03; A11CC01; A11CC02; A11CC03; A11CC04; V03AE02; V03AE03; V03AE05 | 6 |
| Smoking cessation | N07BA01; N07BA02; N07BA03; N06AX12 | 6 |
| Steroid-responsive disease | H02AB01; H02AB02; H02AB03; H02AB04; H02AB05; H02AB06; H02AB07; H02AB08; H02AB09; H02AB10 | 2 |
| Transplant | L04AA06; L04AA10; L04AA18; L04AD01; L04AD02 | 0 |
| Tuberculosis | J04AC01; J04AC51; J04AM01; J04AM02; J04AM03; J04AM04; J04AM05; J04AM06; J04AM07; J04AM08; J04AM21 | NA |
| Pratt, N.L., et al., *The validity of the Rx-Risk Comorbidity Index using medicines mapped to the Anatomical Therapeutic Chemical (ATC) Classification System.* BMJ Open, 2018. **8**(4): p. e021122. | | |

**Supplementary Table 2** Comparing known-groups validity of different comorbidity levels of CCI and Rx-Risk

|  | **Charlson Comorbidity Index** | | | | **p value** | **RxRisk Index** | | | | **p value** |
| --- | --- | --- | --- | --- | --- | --- | --- | --- | --- | --- |
|  | No  (n=38) | Low  (n=64) | High  (n=69) | Very High (n=50) |  | No  (n=67) | Low  (n=58) | High  (n=57) | Very High (n=39) |  |
| **EQ-5D-5L** |  |  |  |  |  |  |  |  |  |  |
| VAS, mean (SD) | 65.6 (12.7) | 59.6 (19.2) | 62.5 (15.5) | 58.6 (21.0) | 0.238 | 63.3 (18.6) | 64.3 (12.8) | 57.6 (19.3) | 58.8 (18.8) | 0.113 |
| Index score, mean (SD) | 0.809 (0.2) | 0.804 (0.2) | 0.781 (0.2) | 0.700 (0.3) | **0.031** | 0.820 (0.2) | 0.801 (0.2) | 0.737 (0.2) | 0.708 (0.3) | **0.018** |
| Level sum score, mean (SD) | 9.5 (3.3) | 9.8 (3.4) | 10.1 (3.7) | 11.2 (4.1) | 0.105 | 9.2 (3.3) | 10.0 (3.3) | 10.8 (3.9) | 11.1 (4.2) | **0.035** |
| Dimensions |  |  |  |  |  |  |  |  |  |  |
| Mobility, n (%) | 25 (65.8) | 45 (70.3) | 48 (69.6) | 39 (78.0) | 0.620 | 46 (68.7) | 43 (74.1) | 38 (66.7) | 30 (76.9) | 0.650 |
| Self-care, n (%) | 23 (60.5) | 37 (57.8) | 45 (65.2) | 26 (52.0) | 0.535 | 37 (55.2) | 37 (63.8) | 34 (59.7) | 23 (59.0) | 0.813 |
| Usual activities, n (%) | 26 (68.4) | 49 (76.6) | 53 (76.8) | 37 (74.0) | 0.781 | 47 (70.2) | 46 (79.3) | 44 (77.2) | 28 (71.8) | 0.627 |
| Pain/ discomfort, n (%) | 24 (63.2) | 37 (57.8) | 39 (56.5) | 38 (76.0) | 0.136 | 34 (50.8) | 36 (62.1) | 40 (70.2) | 28 ( 71.8) | 0.078 |
| Anxiety/depression, n (%) | 15 (39.5) | 30 (46.9) | 35 (50.7) | 26 (52.0) | 0.645 | 27 (40.3) | 27 (46.6) | 30 ( 52.6) | 22 ( 56.4) | 0.357 |
| **Functional impairment (BADL), mean (SD)** | 5.3 (2.0) | 5.9 (2.3) | 5.7 (2.3) | 5.2 (2.2) | 0.312 | 5.4 (2.1) | 5.4 (2.6) | 5.5 (2.4) | 6.0 (2.3) | 0.547 |
| Cognitive status (MMSE), mean (SD) | 18.4 (8.4) | 18.2 (7.1) | 18.5 (6.0) | 19.6 (6.5) | 0.714 | 18.1 (7.0) | 18.6 (8.3) | 19.2 (5.5) | 18.8 (6.4) | 0.829 |
| **Health resource utilization** |  |  |  |  |  |  |  |  |  |  |
| GP consultations, mean (SD) | 1.8 (0.9) | 2.2 (1.9) | 2.0 (1.2) | 2.2 (1.2) | 0.429 | 2.1 (1.5) | 2.1 (1.7) | 2.0 (1.1) | 2.1 (1.2) | 0.981 |
| Hospitalization, n (%) | 8 (21.1) | 12 (18.8) | 16 (23.2) | 14 (28.0) | 0.696 | 10 (14.9) | 11 (19.0) | 18 (31.6) | 11 (28.2) | 0.111 |
| Abbreviations: *B-ADL* Bayer-Activities of Daily Living Scale; *GP* general practitioner; *MMSE* Mini-Mental State Examination; *SD* standard deviation; *VAS* visual analog scale  **Bold values indicate P< 0.05** | | | | | | | | | | |

**Supplementary Table 3** Post hoc power and required sample size calculation for the correlation and regression analyses

|  | **Post hoc power analysis (N=221)** | | | |
| --- | --- | --- | --- | --- |
|  | **Correlation analysis** | | **Regression analysis** | |
|  | **Power**  **(based on observed effect)** | **Sample Size**  **(required for sufficient power)** | **Power**  **(based on observed (pseudo) R²** | **Sample Size**  **(required for sufficient power)** |
| **Health-related Quality of Life** |  |  |  | |
| EQ-5D-5L VAS | 0.4882 | N= 463 | 0.9985 | N=73 |
| EQ-5D-5L index score | 0.8970 | N= 168 | 1.0000 | N=23 |
| EQ-5D-5L Level sum score | 0.8267 | N=207 | 1.0000 | N=18 |
| EQ-5D-5L Dimensions |  |  |  | |
| *Mobility* | 0.7274 | N=264 | 1.0000 | N=32 |
| *Self-care* | 0.2804 | N=906 | 0.9994 | N=66 |
| *Usual activities* | 0.4823 | N=470 | 0.9985 | N=73 |
| *Pain/ discomfort* | 0.7172 | N=270 | 0.9985 | N=73 |
| *Anxiety/depression* | 0.5719 | N=377 | 0.9624 | N=125 |
| **Functional impairment (B-ADL)** | 0.3007 | N=832 | 1.0000 | N=10 |
| **Cognitive status (MMSE)** | 0.0830 | N=6,054 | 1.0000 | N=12 |
| **Health resource utilization** |  |  |  | |
| GP consultations | 0.0811 | N=6,405 | 0.3186 | N=779 |
| Hospitalization | 0.5778 | N=372 | 1.0000 | N=30 |
| Abbreviations: *B-ADL* Bayer-Activities of Daily Living Scale; *GP* general practitioner; *MMSE* Mini-Mental State Examination; *SD* standard deviation; *VAS* visual analog scale  shadings indicate power ≥ 0.8 | | | | |
